# Impaired anandamide/palmitoylethanolamide signaling in hippocampal glutamatergic neurons alters synaptic plasticity, learning, and emotional responses

**DOI:** 10.1038/s41386-018-0274-7

**Published:** 2018-11-15

**Authors:** Tina Zimmermann, Julia C. Bartsch, Annika Beer, Ermelinda Lomazzo, Stephan Guggenhuber, Maren D. Lange, Laura Bindila, Hans-Christian Pape, Beat Lutz

**Affiliations:** 1 Institute of Physiological Chemistry, University Medical Center of the Johannes Gutenberg University Mainz, Duesbergweg 6, 55128 Mainz, Germany; 2 Institute of Physiology I, Westfälische Wilhelms-University Münster, Robert-Koch-Str. 27a, 48149 Münster, Germany; 3 German Resilience Center (DRZ), University Medical Center of the Johannes Gutenberg University Mainz, Langenbeckstr. 1, 55131 Mainz, Germany

**Keywords:** Long-term potentiation, Anxiety, Cellular neuroscience

## Abstract

Endocannabinoid signaling via anandamide (AEA) is implicated in a variety of neuronal functions and considered a promising therapeutic target for numerous emotion-related disorders. The major AEA degrading enzyme is fatty acid amide hydrolase (FAAH). Genetic deletion and pharmacological inhibition of FAAH reduce anxiety and improve emotional responses and memory in rodents and humans. Complementarily, the mechanisms and impact of decreased AEA signaling remain to be delineated in detail. In the present study, using the Cre/loxP system combined with an adeno-associated virus (AAV)-mediated delivery system, FAAH was selectively overexpressed in hippocampal CA1-CA3 glutamatergic neurons of adult mice. This approach led to specific FAAH overexpression at the postsynaptic site of CA1-CA3 neurons, to increased FAAH enzymatic activity, and, in consequence, to decreased hippocampal levels of AEA and palmitoylethanolamide (PEA), but the levels of the second major endocannabinoid 2-arachidonoyl glycerol (2-AG) and of oleoylethanolamide (OEA) were unchanged. Electrophysiological recordings revealed an enhancement of both excitatory and inhibitory synaptic activity and of long-term potentiation (LTP). In contrast, excitatory and inhibitory long-term depression (LTD) and short-term synaptic plasticity, apparent as depolarization-induced suppression of excitation (DSE) and inhibition (DSI), remained unaltered. These changes in hippocampal synaptic activity were associated with an increase in anxiety-like behavior, and a deficit in object recognition memory and in extinction of aversive memory. This study indicates that AEA is not involved in hippocampal short-term plasticity, or eLTD and iLTD, but modulates glutamatergic transmission most likely via presynaptic sites, and that disturbances in this process impair learning and emotional responses.

## Introduction

Alterations in synaptic plasticity have been associated with the occurrence of emotional disorders (anxiety, depression) and impairment in memory processes [1, 2]. Alterations in endocannabinoid system activity are implicated in dysregulated emotional and cognitive responses [3]. Given this observation, endocannabinoid signaling via anandamide (AEA) has been proposed to be a promising therapeutic target for a variety of emotion-related disorders [4]. AEA was also shown to regulate synaptic processes, such as long-term depression (LTD) through cannabinoid CB1 receptor (CB1) and the transient receptor potential vanilloid 1 (TRPV1) (reviewed in [3]), long-term potentiation (LTP) [5], and depolarization-induced suppression of excitation (DSE) [6]. AEA synthesis in neurons was reported to occur both in presynaptic and postsynaptic sites [7]. In contrast to AEA synthesis, AEA degradation is much better understood. The main AEA degrading enzyme is fatty acid amide hydrolase (FAAH), an integral membrane serine hydrolase highly expressed in the brain and located in somata and dendrites of neurons, postsynaptically opposed to axon fibers expressing CB1 receptor [8, 9]. Along with AEA, FAAH also hydrolyzes a number of acylethanolamides (NAEs) [10], including the endogenous bioactive lipid palmitoylethanolamide (PEA) [11], known for its anti-inflammatory and neuroprotective properties [12].

Genetic deletion or pharmacological inhibition of FAAH exerts anxiolytic, antidepressant and analgesic properties in rodents [13–17]. Moreover, complete FAAH knockout mice or mice treated with a FAAH inhibitor display facilitated acquisition of memory and extinction learning [18]. However, the mechanisms by which altered FAAH activity and thereby changed endogenous AEA regulate these processes are not well understood, considering that endocannabinoid-mediated effects at the synaptic level are tightly dependent on the type of neurons and tissue where they are released and/or where their targets (e.g., CB1 receptor) are located. Indeed, complete FAAH knockout mice or FAAH blockers do not provide the possibility to discriminate cell type-specific AEA signaling. Despite the well-documented FAAH-mediated modulation of hippocampal functions, to date, cell type-specific FAAH functions have not yet been reported. Here, we hypothesized that a selective depletion of AEA signaling at hippocampal glutamatergic neurons affects distinct forms of synaptic plasticity associated with behavioral alterations in stress-related emotional response.

Thus, we generated a mouse model in which adeno-associated virus (AAV)-mediated overexpression of FAAH was selectively achieved in the CA1-CA3 pyramidal neurons of the hippocampus, leading to increased expression and enzymatic activity of FAAH, and decreased AEA levels. Efficient spreading of the virus was reached to ensure FAAH overexpression in all hippocampal CA1-CA3 glutamatergic pyramidal neurons. We then investigated whether impaired AEA signaling in FAAH-overexpressing mice leads to alterations of endocannabinoid-regulated synaptic plasticity (LTP, LTD, DSE, DSI), emotional responses (anxiety, stress coping) and memory processes.

## Materials and methods

### Animals

Male mice (2–3 months old) were housed in temperature-controlled and humidity-controlled rooms with a 12-h light-dark cycle and food provided ad libitum. Female mice were not included in this study as animal experimental approval was only for males. Furthermore, extending the studies on female mice would substantially increase the overall number of required animals and experimental procedures, even more so as hormonal changes during the estrous cycle would have to be taken into consideration, which would jeopardize the 3R requirement. All experiments were performed according with the European Community’s Council Directive of 22 September 2010 (2010/63EU) and approved by the respective agency of the State Rhineland-Palatinate (Landesuntersuchungsamt) (permit numbers 23 177-07/G 13-01-021 and 23 177-07/G 17-1-028). Adult NEX-Cre mice (C57BL/6N background) were used to drive expression of Cre recombinase in hippocampal pyramidal neurons [19–21]. NEX-Cre-CB1f/f mice [21] were used to measure basal endocannabinoid levels. Male NEX-Cre and wild-type littermates injected with AAV were used for behavioral tests. Genotyping was performed before and after experiments. FAAH-deficient mice were bred as previously described [16].

### DNA construct and adeno-associated virus

The N-terminally hemagglutinin (HA)-tagged mouse FAAH cDNA with 4 additional amino acids (Glu-Phe-Asp-Asn) between HA and the first codon of FAAH was cloned into the adeno-associated virus (AAV) plasmid containing the cytomegalovirus enhancer/chicken ß-actin (CAG) promoter [20], the woodchuck hepatitis virus posttranscriptional regulatory element (WPRE), and the bovine growth hormone polyadenylation sequence (pA) flanked by AAV2 inverted terminal repeats. A transcriptional Stop cassette flanked by loxP sites was inserted downstream the CAG promoter to allow Cre recombinase-dependent transgene expression of HA-FAAH after injection into a Cre-expressing transgenic mouse line. Chimeric AAV serotype 1/2 vectors were produced and genomic titers determined using the Applied Biosystems ABI 7500 real time PCR cycler as described before [22].

### Cell culture and transfection of HEK cells

HEK293 cells were CaCl_2_ transfected with either CBA-zerotrans-HA-FAAH-WPRE-bGH plasmid together with the HA-NLS-Cre plasmid, or as control, HA-NLS-Cre plasmid. After 60 h, cells were harvested.

### AAV injection

Mice were anesthetized by intraperitoneal injection of fentanyl (0.05 mg/kg, Janssen), midazolam (5 mg/kg, Hameln) and medetomidin (0.5 mg/kg, Zoetis). AAV (1 µl; 1.3 × 10^12^ tu/ml) was injected bilaterally into the dorsal and ventral hippocampus at the following coordinates from bregma: AP: −2.0 mm, ML: ± 2.0 mm, DV: −2.0 mm and AP: −3.1 mm, ML: ± 3.0 mm, DV: −3.5 mm, using a microprocessor-controlled minipump with 34 G beveled needles (World Precision Instruments) at a rate of 200 nl/min. Anesthesia was antagonized by intraperitoneal injection of flumazenil (0.5 mg/kg, Hikma), naloxon (1.2 mg/kg, Ratiopharm) and atipamezol (2.5 mg/kg, Zoetis). Mice received a subcutaneous injection of buprenorphine (0.05 mg/kg), and 0.9% saline to compensate fluid loss and were kept on a heating plate at 37 °C overnight and constantly monitored to ensure recovery.

### Immunohistochemistry

Immunohistochemical analysis in coronal brain sections (30 µm) was carried out as previously described [20]. For details, see Supplementary Information.

### Western blot

Dissected hippocampi were processed as previously reported [23]. For details, see Supplementary Information.

### Endocannabinoid extraction and liquid chromatography–mass spectrometry

Lipid extractions and determination of AEA, PEA, OEA, 2-AG, and AA concentrations were performed as previously described [24]. For details, see Supplementary Information.

### FAAH activity assay

FAAH activity assay was carried out as previously reported [25]. For details, see Supplementary Information.

### Electrophysiology

Mice were decapitated under deep isoflurane anesthesia (2.5% in O_2,_ Abbott, Wiesbaden, Germany) and horizontal slices (300 μm) containing hippocampus and entorhinal cortex were prepared. Whole-cell patch-clamp recordings in voltage-clamp mode were obtained from CA1 pyramidal cells (details reported in Supplementary Information). Drugs were purchased from Abcam plc (Cambridge, UK). Postsynaptic currents (PSCs) were evoked by a bipolar tungsten stimulation electrode placed locally in stratum radiatum. 2-(3-Carboxypropyl)-3-amino-6-(4 methoxyphenyl) pyridazinium bromide (Gabazine, 10 μM), CGP55845 hydrochloride (2.5 μM), DL-2-Amino-5-phosphonopentanoic acid sodium salt (AP5, 25 μM), or 6,7-Dinitroquinoxaline-2,3-dione disodium salt (DNQX, 10 μM) were added to the extracellular solution to block GABAergic or glutamatergic postsynaptic currents, respectively, and to isolate postsynaptic excitatory (EPSCs) or inhibitory (IPSCs) currents.

In long-term plasticity experiments, PSCs were evoked at a frequency of 0.033 Hz. Stable baseline responses were recorded for 10 min. In LTP experiments and for recording of spontaneous (s) EPSCs, cells were held at −70 mV using a K-gluconate-based intracellular solution. The stimulus intensity was set to evoke EPSC amplitudes of 30–50% of the maximum response during baseline recordings. In excitatory LTD (eLTD) experiments, cells were held at -70 mV, and the stimulus intensity was set to evoke EPSC amplitudes of 50% of the maximum response during baseline recordings. In inhibitory LTD (iLTD) experiments and for recording of spontaneous (s) IPSCs, neurons were held at +10 mV using a Cs-methanesulfonate-based intracellular solution. The stimulus intensity was set to evoke IPSC amplitudes of 50–70% of the maximum response during baseline recordings. Changes in synaptic strength were measured for 30 min after induction. Long-term plasticity was calculated by averaging the responses collected during 36–40 min (LTP or iLTD) or 45–50min (eLTD) of each experiment. All individual cells recorded were included into analysis and no arbitrary threshold was set to define LTP, eLTP, or iLTD. Detection and analysis of sEPSCs and sIPSCs was done offline using Mini Analysis program (Version 6.0.7, Synaptosoft Inc., Fort Lee, NJ, USA).

In short-term plasticity experiments, cells were clamped at a holding potential of −70 mV. After recording of stable baseline responses (stimulation frequency 0.2 Hz), depolarization-induced suppression of excitation (DSE) or inhibition (DSI) was tested by application of a depolarization step (−70 to 0 mV, 10 s). Additional information is provided in Supplementary Information.

### Behavioral assays

All tests were performed during the light phase by an experimenter blind to the animal genotype. Trials were video-recorded and analyzed with Noldus Ethovision XT software (Noldus, Wageningen, Netherlands). Each test was performed on a different day in the following order: light/dark test, open field, spatial object recognition test, forced swim test (FST), and passive avoidance test. Light/dark, open field, and passive avoidance tests were carried out as previously described [23]. FST was performed as reported before [26]. The spatial object recognition test was performed in a Y-maze as previously described [27, 28].

### Data analysis

Data were analyzed with GraphPad Prism 4.0, 6.01, or 7.0 (GraphPad Software, La Jolla, CA, USA). Lipid measurement data were analyzed by one-way analysis of variance (ANOVA) followed by Tukey’s multiple comparison test. FAAH activity was analyzed by the Michaelis-Menten equation to determine the Vmax and Km constant, and difference between groups was assessed by one-way ANOVA. Appropriate (paired or unpaired) two-tailed Student’s *t*-test were used to analyze normally distributed data for western blot, passive and active membrane properties, sEPSC and sIPSC kinetics, LTP, eLTD, iLTD, CV-index, and behavioral results. Differences in distributions of sEPSC/sIPSC amplitudes and inter-event intervals were statistically tested using Kolmogorov-Smirnov test. Passive avoidance data, PPI, DSE, and DSI were analyzed by two-way repeated measures ANOVA for ‘time’ and ‘genotype’. ‘Time’ was used as repeated factor and refers to the time points when the test was performed. ANOVA was followed by Bonferroni’s or Sidak’s multiple comparison test to carry out comparison between groups at each specific time point. Data are presented as mean ± SEM and considered significant at a *p*-value < 0.05. If not mentioned in the text, a detailed description of the statistical analyses is reported in Supplementary Tables S1, S2, S3 and S4.

## Results

### In vitro overexpression of FAAH in HEK293 cells

In order to direct FAAH expression to a specific cell population, we generated a Cre recombinase-inducible FAAH overexpression construct. A transcriptional Stop cassette flanked by two loxP sites was placed in front of the FAAH transgene. To differentiate between endogenous and transgene FAAH, a human influenza hemagglutinin (HA) epitope was fused to the N-terminus of FAAH. Co-transfection of Stop-FAAH and Cre-expressing plasmids in HEK293 cells resulted in excision of the transcriptional Stop cassette and initiation of transcription of HA-FAAH. Decreased levels of AEA, PEA and AA in HEK293 cells 60 h after transfection were measured (Fig. S1). Indeed, expression of FAAH led to a significant reduction of AEA (0.55 ± 0.02 ng/g protein; *p* = 0.0001) compared to Cre expression control (1.49 ± 0.07 ng/g protein) and to a significant reduction of PEA (0.10 ± 0.00 µg/g protein; *p* = 0.001) compared to control (0.17 ± 0.01 µg/g protein). Notably, 2-AG levels did not differ between groups. The AA level was also significantly (*p* = 0.017) reduced in FAAH-expressing cells (12.66 ± 1.82 µ/g protein) compared to Cre-expressing control (18.50 ± 0.91 µg/g protein; *n* = 6).

### In vivo overexpression of FAAH specifically in hippocampal glutamatergic neurons

**Fig. 1:**
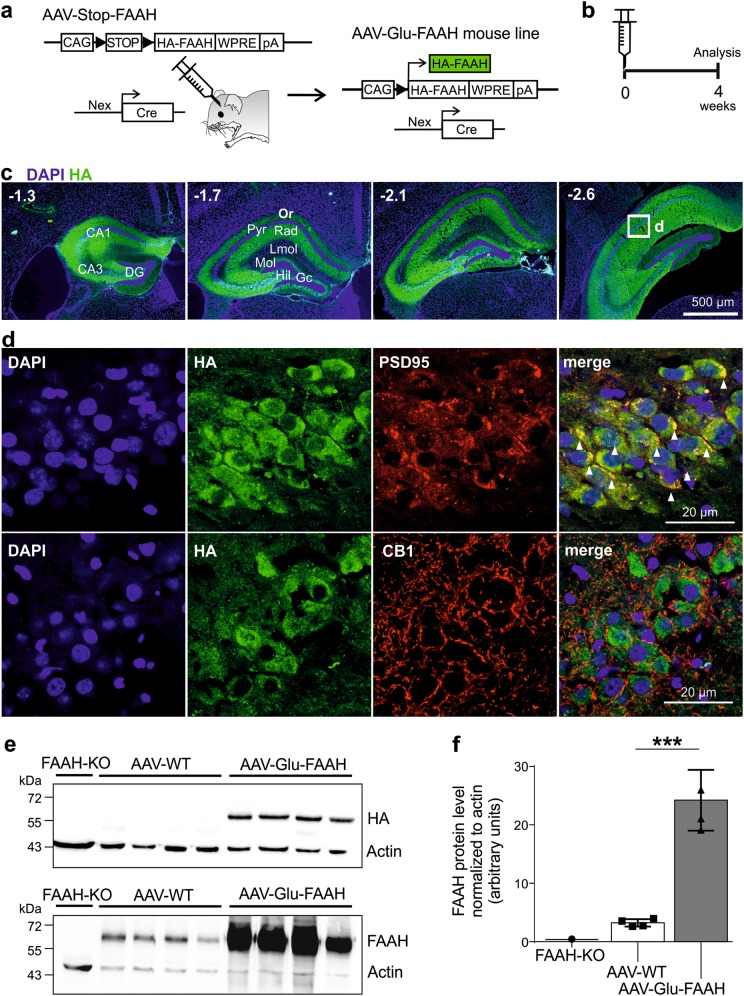
FAAH overexpression in hippocampal CA1-CA3 pyramidal neurons. **a** Schematic diagram showing the experimental strategy for FAAH overexpression. AAV-FAAH contained a ubiquitous promoter (CAG), a loxP flanked (black triangles) transcriptional Stop cassette (STOP), and HA-FAAH encoding sequence. AAV-FAAH was injected into dorsal and ventral hippocampus of Nex-Cre mice. The stop cassette was excised in a Cre-dependent manner, allowing expression of HA-FAAH, termed AAV-Glu-FAAH. WPRE woodchuck hepatitis virus posttranscriptional regulatory element; pA polyadenylation signal. **b** Timeline of experiment. Mice were analyzed 4 weeks post AAV injection. **c** Coronal sections of the injected hippocampus of AAV-Glu-FAAH mice immunostained for HA, displaying the rostrocaudal extent of AAV-mediated HA-tagged FAAH expression. Numbers indicate the distance from bregma. CA1 cornu ammonis region 1, CA3 cornu ammonis region 3, DG dentate gyrus, GC granule cell layer, Hil hilar region, LMol stratum lacunosum-moleculare, Mol stratum moleculare, Or stratum oriens, Pyr pyramidal cell layer, Rad stratum radiatum. **d** HA and postsynaptic density marker 95 (PSD95) colocalization (upper panels; white triangles); HA and presynaptic CB1 receptor immunostaining in the CA1 region (bottom panels). **e** Western blot analysis of homogenized hippocampi from FAAH knockout (FAAH-KO), wild-type control (AAV-WT) and FAAH overexpressing mice (AAV-Glu-FAAH) revealed a specific FAAH signal at 63 kDa in wild-type and transgenic samples, and exclusive transgene (HA) expression in AAV-Glu-FAAH mice. **f** Quantification of FAAH protein levels in hippocampal homogenates showed more than seven-fold increase in transgenic mice (AAV-Glu-FAAH) as compared to wild-type controls (AAV-WT) (*n* = 4). ***p < 0.001, Student´s *t*-test. Data are represented as mean ± SEM. Statistical analysis details are reported in Supplementary Table S2.

Overexpression of FAAH exclusively in hippocampal CA1-CA3 glutamatergic neurons was achieved by injecting AAV-Stop-FAAH bilaterally into the dorsal and ventral hippocampus of transgenic Nex-Cre mice, which express Cre recombinase in CA1-CA3 pyramidal neurons, but not in granule cells of dentate gyrus. The transcriptional Stop cassette was excised, enabling the transcription of HA-FAAH (Fig. 1a). Animals overexpressing FAAH in glutamatergic neurons were named AAV-Glu-FAAH and their control littermates AAV-WT. Four weeks after AAV injection, animals were analyzed (Fig. 1b). HA-immunostaining revealed a homogeneous distribution of HA throughout the entire hippocampus, as exemplified for the dorsal hippocampus with overexpression in the stratum oriens, stratum radiatum, and the inner third of the molecular layer (Fig. 1c). To identify the cellular localization of HA-FAAH, co-immunostaining of HA and the postsynaptic density marker 95 (PSD95) revealed HA-FAAH expression at the postsynaptic side and in the cytosol (Fig. 1d). Immunostaining for CB1 receptor, which is presynaptically located, revealed no overlap between HA and CB1 receptor, indicating that HA-FAAH is postsynaptically expressed at endogenous sites. Western blot analysis of hippocampal lysates using antibodies against HA revealed that HA-FAAH was exclusively expressed in AAV-Glu-FAAH mice, whereas no signal was detected in control AAV-WT and FAAH-deficient mice (FAAH-KO) (Fig. 1e). FAAH protein levels were highly elevated in AAV-Glu-FAAH mice compared to AAV-WT mice, as assessed with an anti-FAAH antibody. Quantification of endogenous FAAH and transgenic HA-FAAH revealed a 7-fold higher expression of HA-FAAH (24.21 ± 2.60 arbitrary units; *p* = 0.0002) compared to endogenous FAAH levels (3.21 ± 0.32 arbitrary units; *n* = 4) (Fig. 1f). Statistical details are reported in Supplementary Table S2.

### Overexpression of FAAH in hippocampal glutamatergic neurons leads to increased degradation of AEA and PEA

**Fig. 2:**
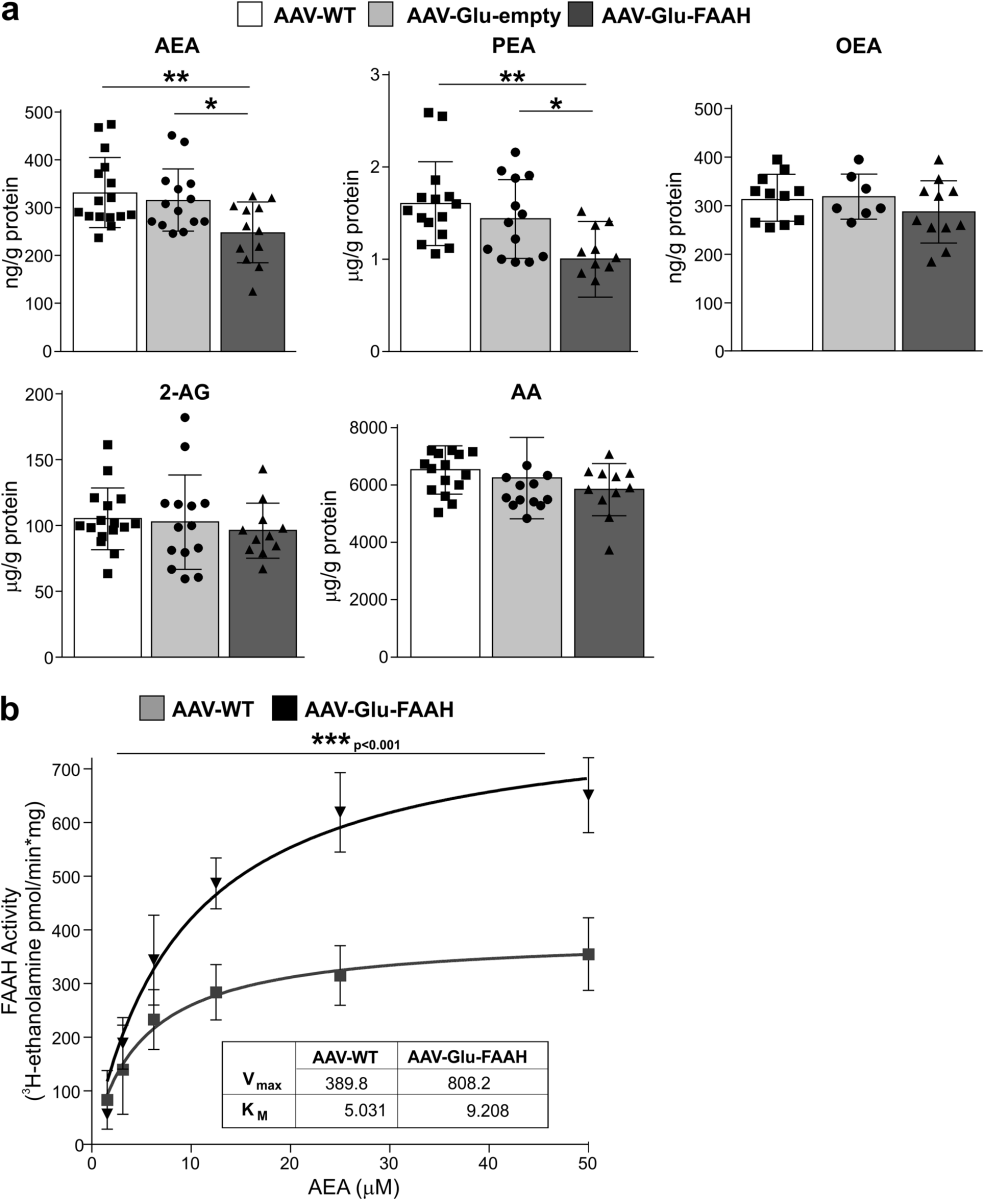
FAAH overexpression in hippocampal CA1-CA3 pyramidal neurons leads to decreased anandamide (AEA) and palmitoylethanolamide (PEA) levels. **a** AEA and PEA levels were significantly lower in AAV-Glu-FAAH samples (*n* = 11-12) as compared to control AAV-WT animals (*n* = 15-16) and AAV-empty injected mice (*n* = 12-14), whereas oleoylethanolamide (OEA), arachidonic acid (AA), and 2-arachidonoylglycerol (2-AG) levels were not altered. **b** FAAH activity is enhanced upon FAAH overexpression in hippocampus. Along increasing amounts of substrate [^3^H]-AEA (0–50 μM range), FAAH activity was increased in AAV-Glu-FAAH (*n* = 3) as compared to AAV-WT (*n* = 3) mice as indicated by the increase in the maximal amount of product [^3^H-ethanolamine], and the statistically significant increase in *V*_max_ (inset). No significant change in K_m_ constant was found. Data are expressed as amount of product [^3^H-ethanolamine] (pmol) generated over reaction time (min) and protein amount (mg). *p < 0.05, **p < 0.01, ***p < 0.001 one-way ANOVA with Tukey’s multiple comparison test. *V*_max_ and *K*_m_ values were obtained by using a Michaelis-Menten equation-based analysis. Values are expressed as means ± SEM. Statistical analysis details are reported in Supplementary Table S3.

Cell type-specific overexpression of FAAH led to a significant reduction of AEA (Fig. 2a) in AAV-Glu-FAAH animals (248.30 ± 18.39 ng/g protein; *n* = 12) compared to control AAV-Glu-empty injected mice (315.90 ± 17.42 ng/g protein; *p* = 0.013; *n* = 16) and AAV-WT mice (331.60 ± 18.37 ng/g protein; *p* = 0.004; *n* = 14). PEA levels were also significantly lower in AAV-Glu-FAAH mice (1.00 ± 0.12 µg/g protein; *n* = 11) than in AAV-WT mice (1.60 ± 0.11 µg/g protein; *p* = 0.001; *n* = 15) and AAV-Glu-empty injected mice (1.43 ± 0.11 µg/g protein; *p* = 0.018; *n* = 13). No difference in OEA, 2-AG and AA levels was found. Statistical details are reported in Supplementary Table S3.

### Overexpression of FAAH in hippocampal glutamatergic neurons leads to enhanced FAAH activity

The FAAH degradation product [^3^H-ethanolamine] (Fig. 2b) was increased in AAV-Glu-FAAH compared to AAV-WT mice, indicating enhanced FAAH enzyme activity in FAAH-overexpressing animals (*p* < 0.001; *n* = 3). A statistically significant increase in the *V*_max_ was measured in AAV-Glu-FAAH (808.2 ± 83.11 pmol/mg protein; *p* = 0.0113) compared to AAV-WT mice (389.8 ± 46.43 pmol/mg protein), indicating that at saturating substrate concentrations (µM range) AAV-Glu-FAAH mice show elevated FAAH activity rate compared to controls. No difference in the *K*_m_ constant was found. Our data confirm the reliability of the AAV-mediated approach used here, as no altered FAAH activity (Fig. 2b) and/or endocannabinoid levels (Fig. 2a) were found in empty vector-injected mice, which may occur, for instance, when using a lentivirus-mediated approach due to potential lack of construct specificity [29]. Statistical details are reported in Supplementary Table S3.

### Elevated FAAH levels in hippocampal pyramidal neurons increase LTP, but not eLTD, iLTD, DSE, or DSI in CA1 pyramidal cells

**Fig. 3:**
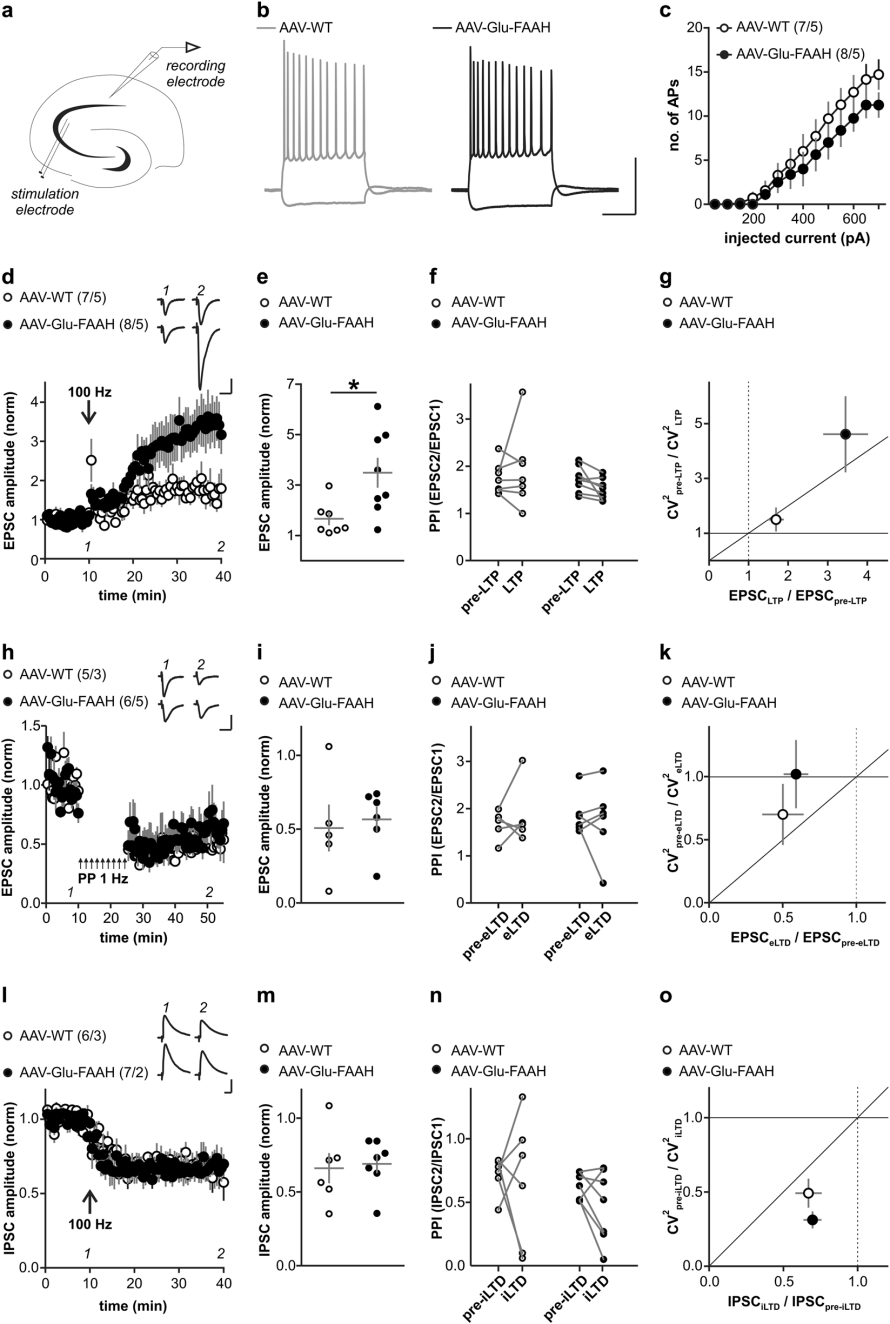
Long-term plasticity at hippocampal CA3-CA1 synapses. **a** Schematic drawing illustrates placement of stimulation and recording electrodes in the hippocampal slice. **b** Discharge pattern of CA1 neurons upon depolarizing (500 pA) and hyperpolarizing (−250 pA) current pulses. Scale bars: 50 mV, 200 ms. **c** Number of generated action potentials (APs) plotted against positive current injection (in pA) in CA1 pyramidal cells of AAV-WT and AAV-Glu-FAAH mice. **d**, **e** LTP in CA1 following high-frequency stimulation (HFS, 100 Hz) is significantly enhanced in AAV-Glu-FAAH mice compared to AAV-WT mice (unpaired *t* test, *p < 0.05). **d** Inset shows sample EPSC traces at indicated time points before and after HFS, scale bars: 100 pA, 25 ms. **f** PPI does not change after induction of LTP. **g** Analysis of the coefficient of variation (CV^2^) plotted against mean EPSC ratios indicates a presynaptic mechanism involved in LTP in AAV-Glu-FAAH mice and a postsynaptic one in AAV-WT mice. **h, i** Excitatory LTD (eLTD) at CA3-CA1 synapses following low-frequency stimulation (LFS, 900 paired pulses, 1 Hz) is unchanged in AAV-Glu-FAAH mice compared to AAV-WT mice. **h** Inset shows sample EPSC traces at indicated time points before and after LFS, scale bars: 100 pA, 25 ms. **j** PPI does not change after induction of eLTD. **k** Analysis of CV^2^ plotted against mean EPSC ratios point to a postynaptic mechanism involved in eLTD in both genotypes. **l**, **m**. Both AAV-WT and AAV-Glu-FAAH mice showed intact inhibitory LTD (iLTD) at CA3-CA1 synapses. In (l), inset shows sample IPSC traces at indicated time points before and after HFS, scale bars: 200 pA, 25 ms. **n** PPI does not change after induction of iLTD. **o** Analysis of CV^2^ plotted against mean IPSC ratios indicates a presynaptic mechanism involved in iLTD in mice of both genotypes. In **e**, **i**, **m**, scatter plots and corresponding mean ± SEM represent amplitudes recorded after plasticity induction (LTP and iLTD: min 36–40; eLTD: min 46–50). In **c**, **d**, **h**, **l**, numbers indicate the number of recorded cells/animals.

Reduced AEA levels could compromise endocannabinoid-mediated depression of synaptic transmission and lead to functional effects at the synaptic level. Therefore, we analyzed basic synaptic properties by whole-cell recordings of sEPSCs and sIPSCs from CA1 pyramidal neurons. AAV-Glu-FAAH mice displayed shorter inter-event intervals and slightly smaller amplitudes of sEPSCs compared to AAV-WT mice (inter-event intervals: AAV-WT: 2.09 ± 0.15 s, *n* = 7 cells from 5 mice, AAV-Glu-FAAH: 1.65 ± 0.14 s, *n* = 8 cells from 5 mice, *p* = 0.0007, Kolmogorov–Smirnov test, amplitudes: AAV-WT: 18.70 ± 0.56 pA, AAV-Glu-FAAH: 16.22 ± 0.39 pA, *p* = 0.01, Kolmogorov-Smirnov test, Fig. S2a-d). Inter-event intervals of sIPSCs were also significantly shorter in AAV-Glu-FAAH mice (AAV-WT: 245 ± 16 ms, *n* = 6 cells from 3 mice, AAV-Glu-FAAH: 251 ± 28 ms, *n* = 7 cells from 2 mice, *p* = 0.0001, Kolmogorov–Smirnov test, Fig. S2e-h). *T*_rise_ and *T*_decay_ of sEPSCs and sIPCSs were unchanged in both genotypes (sEPSC: AAV-WT: *T*_rise_: 1.63 ± 0.17 ms, *T*_decay_ 5.78 ± 0.53 ms; AAV-Glu-FAAH: T_rise_: 1.93 ± 0.13 ms, *T*_decay_ 6.60 ± 0.33 ms; sIPSC: AAV-WT: *T*_rise_: 1.14 ± 0.10 ms, *T*_decay_ 14.33 ± 1.24 ms; AAV-Glu-FAAH: *T*_rise_: 1.07 ± 0.15 ms, *T*_decay_ 13.83 ± 0.83 ms, Fig. S2c, f). Generally, differences in intrinsic membrane properties of CA1 pyramidal cells could contribute to changes in sEPSC and sIPSC. However, no differences in basic passive and active membrane properties of CA1 pyramidal cells were found (AAV-WT: *n* = 7 cells from 5 mice, AAV-Glu-FAAH: *n* = 8 cells from 5 mice, Fig. 3b, c; Supplementary Table S1).

Endocannabinoids can induce long-term and short-term plasticity at both excitatory and inhibitory synapses. Therefore, we recorded evoked EPSCs and IPSCs from CA1 pyramidal cells upon local electrical stimulation in stratum radiatum (Fig. 3a) and tested the effects of FAAH overexpression on different forms of synaptic plasticity.

LTP appeared as a lasting increase in EPSC amplitudes in CA1 pyramidal cells following high-frequent stimulation in stratum radiatum (4 trains of 100 pulses at 100 Hz with 30 s inter-train intervals). In AAV-Glu-FAAH mice, LTP was significantly enhanced compared to AAV-WT mice (AAV-Glu-FAAH: 3.49 ± 0.58 of baseline, *n* = 8 cells from 5 mice; AAV-WT: 1.66 ± 0.25 of baseline, *n* = 7 cells from 5 mice; *p* = 0.017, unpaired *t* test, Fig. 3d, e). Different approaches were used to identify the locus (pre- or postsynaptic) of LTP. The PPI depends on residual Ca^2+^ levels in the presynaptic terminal and investigates the ability of synapses to increase transmitter release upon the second of two closely spaced afferent stimuli [30]. Consistent with a postsynaptic expression of LTP [31], PPI did not change after LTP induction (AAV-WT: from 1.76 ± 0.13 to 1.93 ± 0.31; AAV-Glu-FAAH: from 1.72 ± 0.10 to 1.51 ± 0.07, two-way RM ANOVA, factor time: F_(1, 13)_ = 0.03, *p* = 0.87, Fig. 3f). Changes in coefficient of variation (CV)-index accompanying synaptic plasticity can also be used to distinguish between pre- and postsynaptic mechanisms [32]. Only in AAV-Glu-FAAH mice, LTP went along with changes in CV-index, corroborating involvement of additional presynaptic mechanisms after AEA depletion (CV-index: AAV-WT: from 19.94 ± 7.67 to 27.13 ± 10.83, *p* = 0.25; AAV-Glu-FAAH: from 27.28 ± 8.57 to 76.90 ± 13.30, *p* < 0.01, paired *t* test, Fig. 3g).

In contrast, LTD of excitatory (eLTD) and inhibitory transmission (iLTD), observed as a lasting decrease in either EPSC or IPSC amplitudes upon repetitive stimulation (eLTD: 900 paired pulses with 50 ms inter-pulse interval at 1 Hz, [33]; iLTD: 2 trains of 100 pulses at 100 Hz with 20 s inter-train intervals, [34]), did not differ between animal groups (eLTD: AAV-Glu-FAAH: 0.57 ± 0.09 of baseline, *n* = 6 cells from 5 mice; AAV-WT: 0.51 ± 0.16 of baseline, *n* = 5 cells from 3 mice; *p* = 0.74, unpaired *t* test, Fig. 3h, i; iLTD: AAV-Glu-FAAH: 0.69 ± 0.06 of baseline, *n* = 7 cells from 2 mice; AAV-WT: 0.66 ± 0.10 of baseline, *n* = 6 cells from 3 mice; *p* = 0.81, unpaired *t* test, Fig. 3l, m). In both genotypes, PPI did not change after eLTD or iLTD induction, pointing to a postsynaptic mechanism (eLTD: AAV-WT: from 1.66 ± 0.13 to 1.87 ± 0.26; AAV-Glu-FAAH: from 1.87 ± 0.16 to 1.75 ± 0.29, two-way RM ANOVA, factor time: F_(1, 9)_ = 0.07, *p* = 0.80, Fig. 3j; iLTD: AAV-WT: from 0.72 ± 0.06 to 0.66 ± 0.21; AAV-Glu-FAAH: from 0.62 ± 0.04 to 0.47 ± 0.11, two-way RM ANOVA, factor time: F_(1, 11)_ = 0.81, *p* = 0.39, Fig. 3n). However, iLTD-associated changes in CV-index in both genotypes indicate additional presynaptic mechanisms (CV-index: AAV-WT: from 133.59 ± 38.86 to 77.48 ± 31.32, *p* < 0.05; AAV-Glu-FAAH: from 180.90 ± 29.03 to 53.49 ± 12.99, *p* < 0.001, paired *t* test, Fig. 3o).

**Fig. 4:**
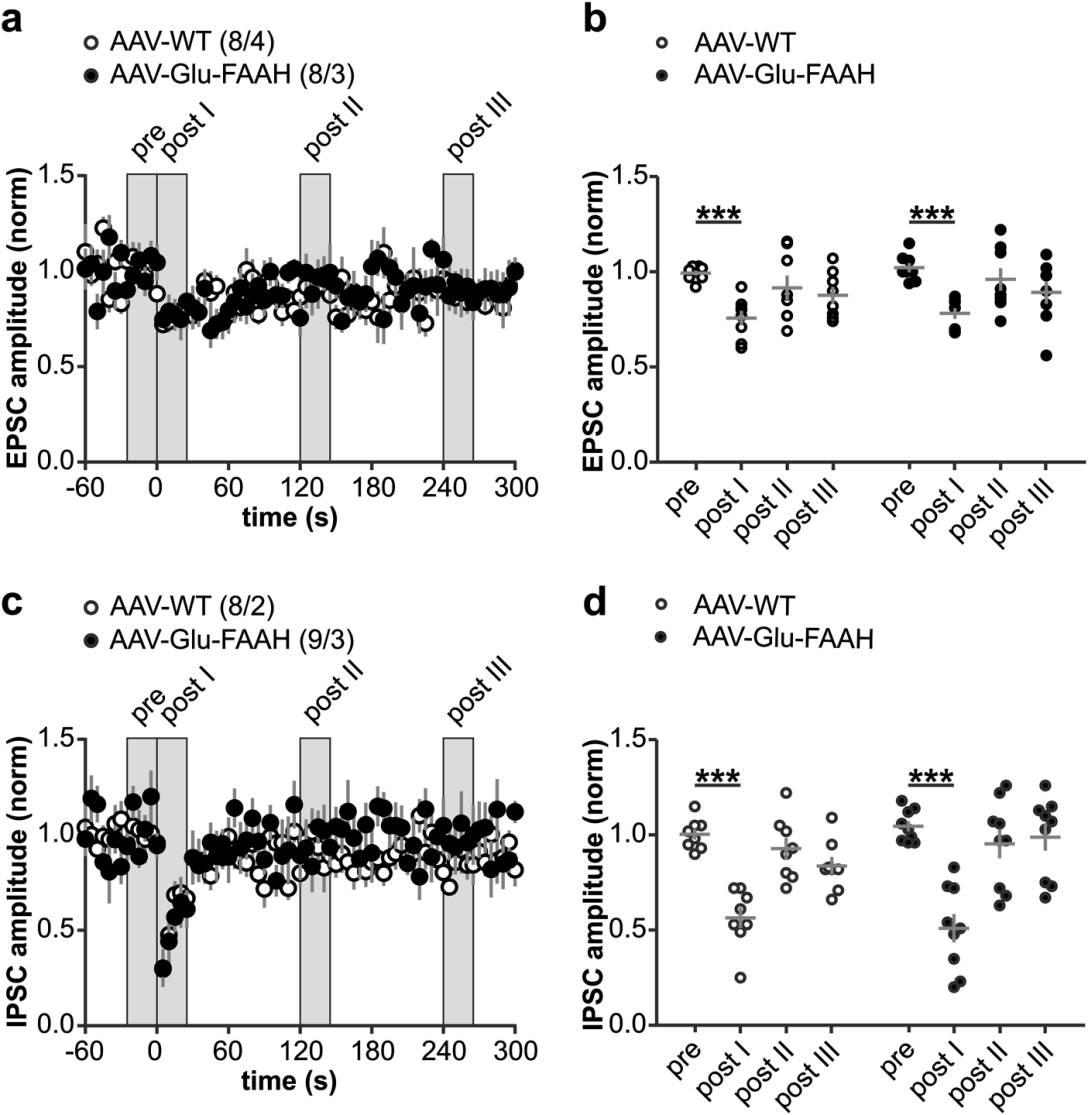
Depolarization-induced suppression of excitation (DSE) and inhibition (DSI) at hippocampal CA3-CA1 synapses. **a**, **c** Upon postsynaptic depolarization (−70 to 0 mV, 10 s duration, at time zero), both AAV-WT and AAV-Glu-FAAH mice showed comparable DSE and DSI. Numbers indicate the number of recorded cells/animals. **b**, **d** Scatter plot and corresponding mean ± SEM of amplitudes recorded immediately before (pre), immediately after (post I), 2 min (post II) and 4 min after depolarization (post III). Repeated measure-ANOVA, factor time in **b**: F_(3, 42)_ = 13.22, *p* < 0.0001, factor time in **d**: F_(3, 45)_ = 36.88, *p* < 0.0001, ***p < 0.001 pre vs. post I, by post hoc Bonferroni.

Endocannabinoid-mediated short-term plasticity is exemplified by phenomena known as DSE and DSI: a brief postsynaptic depolarization leads to transient depression of excitatory or inhibitory transmission (mean of 5 electrically evoked responses immediately after depolarization termed ‘post I’ in Fig. 4). Both DSE and DSI existed in AAV-Glu-FAAH mice, and the magnitude of suppression did not differ from AAV-WT mice (DSE at time point ‘post I’: AAV-Glu-FAAH: 0.78 ± 0.03, *n* = 8 cells from 3 mice; AAV-WT: 0.76 ± 0.04, *n* = 8 cells from 4 mice, RM-ANOVA, factor time in (b): F_(3, 42)_ = 13.22, *p* < 0.0001, ***p < 0.001 pre vs. post I, by post hoc Bonferroni, Fig. 4a, b; DSI at time point ‘post I’: AAV-Glu-FAAH: 0.51 ± 0.07, *n* = 9 cells from 3 mice; AAV-WT: 0.57 ± 0.06, *n* = 8 cells from 2 mice, RM-ANOVA, factor time in (d): F_(3, 45)_ = 36.88, *p* < 0.0001, ***p < 0.001 pre vs. post I, by post hoc Bonferroni, Fig. 4c, d). These results demonstrate that FAAH overexpression leads to increased excitatory and inhibitory synaptic activity and enhanced LTP at glutamatergic CA3-CA1 synapses, while eLTD, iLTD, DSE, and DSI are not compromised.

### Increased FAAH levels in hippocampal glutamatergic neurons affect emotional behavior and hippocampal-dependent memory formation

**Fig. 5:**
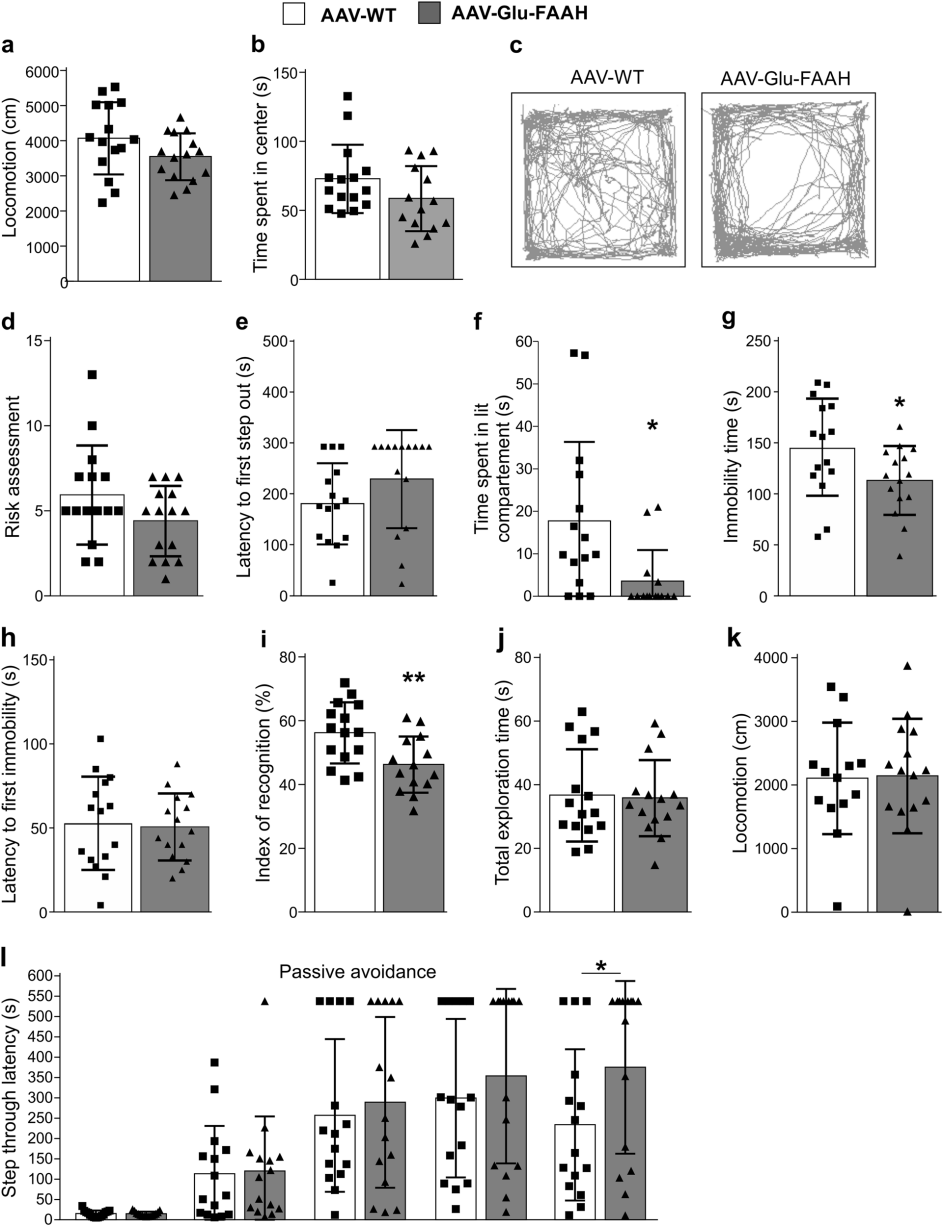
Effect of FAAH overexpression on anxiety-like behavior and memory formation. **a** Animals tested in the open field test showed equal locomotor activity, although (**b**) AAV-Glu-FAAH mice tended to spend less time in the center than AAV-WT mice. **c** Representative movement pattern of individual mice exposed to the open field, showing a center-avoiding behavior in AAV-Glu-FAAH mice. **d** In the light dark test, AAV-Glu-FAAH mice showed unaltered risk assessments, (**e**) no change in latency to first step out, and (**f**) a decrease in time spent in the lit compartment. **g** In the forced swim test, AAV-Glu-FAAH mice were less immobile compared to control, whereas (**h**) the latency to first immobility was unchanged. (**i**) Spatial memory was assessed by the spatial object recognition test. AAV-Glu-FAAH mice showed a decrease in the index of recognition, while **(j**) total exploration time and (**k**) locomotor activity were not affected. **(l)** In the fear-motivated avoidance task AAV-Glu-FAAH mice displayed equal memory acquisition (first two bars), and equal step-through latencies 1 h, 24 h and 1 week after conditioning, but a significant increase in the step-through latencies 2 weeks after conditioning (last two bars). *n* = 15 mice per group. *p < 0.05, **p < 0.01, Student´s *t*-test and two-way repeated measures ANOVA with Sidak’s multiple comparison test. Values are expressed as means ± SEM. Statistical analysis details are reported in Supplementary Tables S2 and S4.

Dysregulation in the signaling of endocannabinoids and subsequent changes in hippocampal synaptic plasticity have been implicated in alterations of emotional behavior and memory tasks. In the open field test, no significant difference in locomotion between WT and FAAH-overexpressing mice was detected (Fig. 5a). Furthermore, both experimental groups spent the same amount of time in the center (Fig. 5b). However, representative traces of mice moving through the open field (Fig. 5c) showed a center-avoiding behavior in the AAV-Glu-FAAH mice compared to control. In the light dark test, AAV-Glu-FAAH mice showed no change in risk assessments (Fig. 5d) or in the latency to the first step out (Fig. 5e), but spent significantly less time in the lit compartment (Fig. 5f, *p* = 0.013) compared to AAV-WT mice. This suggests an increase in anxiety-like behavior in FAAH-overexpressing mice.

In the FST, AAV-Glu-FAAH mice showed reduced immobility time compared to AAV-WT mice (Fig. 5g, *p* = 0.038), suggesting increased struggling as a likely sign of altered stress coping behavior. No difference was found in the latency to first immobility (Fig. 5h).

Modifications of hippocampal function can lead to alterations in learning and memory. To assess differences in spatial memory, the spatial object recognition test was performed. The index of recognition (Fig. 5i) was significantly decreased (*p* = 0.007) in AAV-Glu-FAAH compared to AAV-WT mice, whereas total exploration time (Fig. 5j) and locomotion (Fig. 5k) were unaltered. To assess the impact of FAAH overexpression on the formation of aversive memory, the passive avoidance test was carried out and revealed no changes in the latency to enter the dark chamber during memory acquisition, as well as after 1 h, 24 h, and 1 week post-acquisition. However, 2 weeks after acquisition, AAV-Glu-FAAH mice showed a statistically significant prolonged latency to enter the dark chamber as compared to AAV-WT mice (Fig. 5l, *p* = 0.025; *n* = 15). Statistical details are reported in Supplementary Table S2 and Supplementary Table S4.

## Discussion

Previous investigations have demonstrated that FAAH represents an important pharmacological target for the treatment of a variety of diseases. Pharmacological blockade of the enzyme with selective FAAH inhibitors such as URB597, leading to increased AEA levels, exerts anxiolytic [14, 16, 35], antidepressant [17] and analgesic [36] effects. However, recent clinical developments have demonstrated the necessity to design highly selective FAAH inhibitors, lacking off-target activities which may lead to neurotoxicity [37]. This stresses the importance of a detailed understanding of the mechanisms underlying FAAH activity in the CNS, for further exploring its therapeutic translational potential. In following this need, and in order to understand in detail how FAAH shapes synaptic function and behavior, we overexpressed FAAH specifically in hippocampal glutamatergic neurons, allowing a cell type-specific decrease of AEA signaling. We observed overexpressed HA-FAAH in the cell soma and in postsynaptic compartments of hippocampal CA1-CA3 glutamatergic neurons, coinciding with the endogenous expression sites of FAAH and opposed to presynaptic CB1 receptor expression site [8]. Overexpression of HA-FAAH protein was seven-fold in relation to endogenous FAAH, while FAAH hydrolyzing activity was enhanced two-fold. Consequently, FAAH overexpression led to a 25% decrease of AEA and to a 33% decrease in PEA levels, revealing that FAAH metabolizing activity is specific for AEA and PEA. The lack of proportional change in enzyme overexpression, enzyme activity and change in endocannabinoid levels was previously reported in mice overexpressing monoacylglycerol lipase (MAGL) in hippocampus [23]. This discrepancy can be explained by considering that our approach aims at overexpressing FAAH exclusively in CA1-CA3 pyramidal neurons, whereas determination of FAAH levels and activity was carried out in the whole hippocampal tissue where other sites of FAAH expression exist.

FAAH overexpression led to increased LTP along with reduced cognitive performance and preference for novelty in the hippocampus-dependent spatial object recognition test. Our results reveal an inverse correlation between LTP and short-term memory, suggesting that an abnormally high LTP might diminish selective synapse-specific plasticity required for the encoding of novel information and thereby disturb hippocampal network performance [38]. Enhanced LTP in AAV-Glu-FAAH mice was associated with impaired extinction of aversive memory, which is a phenotypic outcome similarly found in complete CB1 receptor knock-out mice [39]. The decreased AEA signaling in AAV-Glu-FAAH led to reduced extinction, which is congruent with the well-reported necessity of proper AEA signaling for extinction of amygdala-mediated fear [26] and for reversal of learning of spatial memory [18].

A convergent line of evidence indicates that an altered balance between glutamatergic and GABAergic synaptic transmission is a hallmark of anxiety and depression-related disorders [40], and the endocannabinoid system represents one of the key regulatory elements in anxiety-like behavior [3]. Previous works predominantly focused on the application of FAAH inhibitors, which led to anxiolytic-like effects [16]. Conversely, our data show that genetically-induced FAAH overexpression in hippocampal pyramidal neurons elicited anxiety-like behavior. AAV-Glu-FAAH mice also displayed a decrease in immobility time in the FST, which may relate to the anxiogenic phenotype and alteration in stress coping. Reduced immobility in FST has been reported after stress exposure or corticotropin-releasing hormone (CRH) administration [41], and upon application of antidepressant drugs [42] or FAAH inhibitors [43]. Interestingly, mice lacking CB1 receptor in glutamatergic neurons displayed an analogous phenotype of augmented anxiety-like behavior and reduced immobility in FST [44], suggesting that stress coping in response to FST is dependent on CB1 receptors expressed specifically on glutamatergic neurons.

We showed that FAAH-overexpression in glutamatergic hippocampal neurons leads to enhanced excitatory long-term plasticity. This alteration in excitatory drive will also affect neuronal activity in projections from hippocampus, in particular from ventral hippocampus, to brain structures known to be involved in anxiety and fear extinction [45, 46], including medial prefrontal cortex (mPFC), basal amygdala (BA), hypothalamus, and lateral septum. Consequently, dysfunctional excitatory glutamatergic tone e.g., in mPFC and BA may occur and can trigger aversive emotional responses leading to anxiety and emotional disturbances [47]. Consistent with this view, dynamics of AEA signaling in behaviorally challenging situations (e.g., stress, fear learning) have been shown in hippocampus, and e.g., pharmacological interference in AEA signaling can induce alterations in emotional outputs [48, 49].

Investigation of functional effects of FAAH overexpression at the synaptic level revealed no changes in DSE and DSI, which is in line with previous studies, suggesting that 2-AG rather than AEA predominantly mediates these forms of short-term synaptic plasticity [23, 50, 51]. Although FAAH overexpression in cultured excitatory autaptic neurons reduced the duration of DSE [6], we observed that decreased AEA and PEA levels by overexpressing FAAH in vivo did not affect endocannabinoid-mediated DSI/DSE at hippocampal CA1-CA3 synapses. Thus, 2-AG seems to mediate the short-term retrograde control of presynaptic inputs at hippocampal CA3-CA1 synapses in adult mice.

Endocannabinoids also regulate long-term synaptic plasticity by modulating both LTD [3, 52] and LTP [5, 53]. In our study, AAV-Glu-FAAH animals showed intact eLTD and iLTD, but enhanced LTP, suggesting a specific role for AEA in modulating LTP at hippocampal excitatory synapses in adult mice. Our results extend previous findings showing that increased AEA levels upon application of the FAAH inhibitor URB597 impair LTP [5]. Additionally, full CB1-KO mice [54, 55] show enhanced hippocampal  LTP, suggesting that AEA-modulated LTP is CB1 receptor-dependent, although involvement of other receptors (e.g., TRPV1) must be also considered [50]. Importantly, our study shows for the first time that FAAH overexpression in hippocampal glutamatergic neurons increases LTP at excitatory synapses. Changes in CV-index support a presynaptic modulation of this LTP. Notably, overexpression of FAAH also led to increased spontaneous glutamatergic and GABAergic transmission, suggesting involvement of presynaptic sites. Given the glutamatergic nature and presynaptic site of LTP modulation, it is feasible to conclude that the enhanced LTP in AAV-Glu-FAAH animals indicates AEA-mediated regulation of glutamate release. However, as alteration in various neurotransmitter levels may contribute to facilitation of LTP [53], neurotransmitters other than glutamate may be also involved. Conversely, 2-AG may be responsible for modulating hippocampal iLTD, based on evidence that blockade of 2-AG production abolished iLTD at hippocampal CA1-CA3 synapses [34]. High-frequency stimulations, as used in the present study, were shown to elevate 2-AG but not AEA levels in hippocampal slices [56], which further corroborates that 2-AG may be the predominant endocannabinoid modulating LTD at hippocampal inhibitory synapses. Moreover, activation of postsynaptic group I metabotropic glutamate receptors is thought to reduce 2-AG-mediated GABA release [34]. Previous studies on eLTD and endocannabinoids at hippocampal CA3-CA1 synapses have been conducted using juvenile rodents as opposed to adult mice in the present study. In line with our findings, they either point to 2-AG or noladin ether, but not AEA being the endocannabinoid involved in eLTD [57, 58].

2-AG may be responsible for DSI/DSE modulation, although additional unconventional endocannabinoid signaling in AAV-Glu-FAAH mice may be implicated, involving, for instance, cross talk between 2-AG (though CB1) and AEA (though TRPV1), considering the latter being a well-established form of non-retrograde endocannabinoid signaling modulating LTD [59]. It is important to consider that our study uses exclusively male animals. Female and male subjects have a distinct hippocampal inhibitory physiology. Endocannabinoid signaling, and specifically AEA, modulates inhibitory synapses in female rat hippocampus and pharmacological FAAH inhibition promotes suppression of inhibition [60]. These notions highlight the necessity to investigate endocannabinoid signaling-mediated regulation of physiology, behavior and its therapeutic value in the female brain, which thus, will be part of our next future investigations.

Although FAAH favorably hydrolyzes AEA [61], which is approximately ten-fold less abundant than OEA and PEA in the brain [62], FAAH overexpression in glutamatergic neurons promoted AEA and PEA metabolism, but had no effect on OEA levels. The impaired PEA signaling may also have contributed to the functional and behavioral alterations in FAAH-overexpressing mice, likely through PPARs and/or GPR55 pathways. Hippocampal PEA modulates reward and memory in mesolimbic areas through GPR55 receptors with the implication of glutamatergic projections emerging from ventral hippocampus [63]. Genetic deletion of PPARγ triggers anxiety and enhances stress response and PPARγ agonists have anxiolytic properties, although PEA/PPARγ-mediated anxiolysis involves mostly amygdala rather than hippocampus [64]. Differently from previous studies where PEA contribution to anxiety states is addressed by enhancing PEA pathways, our mouse model adopts an AEA/PEA signaling depletion approach, where investigating PEA-PPARγ and PEA-GPR55 pathways is very challenging. Indeed, connecting the functional contributions of PEA and behavioral outcome is not straightforward, as PEA is not the unique endogenous ligand at PPARγ and/or GPR55 receptors. Further studies will have to address the differential functions of AEA and PEA signaling via their respective receptors regarding behavior and synaptic processes.

In conclusion, our study shows that FAAH-mediated depletion of AEA and PEA levels and AEA-mediated synaptic signaling in hippocampal glutamatergic neurons modulates LTP and distinct behaviors in mice. Our experimental strategy using the AAV-stop-FAAH construct represents a useful tool to further elucidate AEA and/or PEA signaling and function in the brain.

## FUNDING AND DISCLOSURE

This work was supported in part by the German Research Foundation (CRC/TR 58 to BL (A04) and HCP (A03, A04), and CRC 1193 to BL). The authors declare no competing interests.

## Electronic supplementary material

Supplemental Information
