# Supplementary material for: Impaired anandamide/palmitoylethanolamide signaling in hippocampal glutamatergic neurons alters synaptic plasticity, learning, and emotional responses
Source: Neuropsychopharmacology. 2018 Nov 15;44(8):1377–88. doi: 10.1038/s41386-018-0274-7 (PMC6784910; doi:10.1038/s41386-018-0274-7)
Supplement: Supplementary file 1 — Supplemental Information [file 41386_2018_274_MOESM1_ESM.docx]

**Supplementary Information for**

**Impaired anandamide/palmitoylethanolamide signaling in hippocampal glutamatergic neurons alters synaptic plasticity, learning and emotional responses**

Tina Zimmermann^*^, Julia C. Bartsch^*^, Annika Beer^*^, Ermelinda Lomazzo, Stephan Guggenhuber, Maren D. Lange, Laura Bindila, Hans-Christian Pape, Beat Lutz

*T.Z., J.C.B., A.B. share first authorship

Corresponding author: Beat Lutz, Institute of Physiological Chemistry, University Medical Center of the Johannes Gutenberg University Mainz, Duesbergweg 6, Mainz, Germany, Email: beat.lutz@uni-mainz.de

**Inventory of Supplementary Information**

**Supplementary Materials and Methods**

- Immunohistochemistry
- Western Blot
- Endocannabinoid Extraction and Liquid Chromatography-Mass Spectrometry
- FAAH activity assay
- Electrophysiology

**Supplementary Figures and Table**

- Supplementary Figure S1: Overexpression of fatty acid amide hydrolase (FAAH) in HEK293 cells leads to increased degradation of anandamide (AEA) and palmitoylethanolamide (PEA).
- Supplementary Figure S2: FAAH overexpression in hippocampal CA1-CA3 pyramidal neurons increases spontaneous glutamatergic and GABAergic transmission at CA3-CA1 synapses.
- Supplementary Table S1: Passive and active membrane properties of CA1 pyramidal neurons.
- Supplementary Table S2: Statistical Data Analyses (referred to Figure 1f and Figure 5)
- Supplementary Table S3: Statistical Data Analyses (referred to Figure 2)
- Supplementary Table S4: Statistical Data Analyses (referred to Figure 5l)

**Supplementary Materials and Methods**

*Immunohistochemistry*: Primary antibodies included mouse anti-postsynaptic density protein 95 (PSD-95, BD Transduction Laboratories, Franklin Lakes, NJ, USA; 1:500), rabbit anti-HA (Santa Cruz Biotechnology, Santa Cruz, CA, USA; 1:500, 1:1000) and rabbit anti-CB1 (Frontier Institute, Hokkaido, Japan, 1:500). The specificity of anti-CB1 antibody was previously validated in our laboratory (Guggenhuber *et al,* 2015). Anti-rabbit IgG AlexaFluor488 (Life Technologies, Eugene, OR, USA; 1:1000) and goat anti-mouse IgG AlexaFluor546 (Life Technologies, Eugene, OR, USA; 1:1000) were used as secondary antibodies. Sections were counterstained with 4’,6-diamidino-2-phenylindole (DAPI) and mounted with Mowiol. Stained brain sections were analyzed with a Leica DM5500 fluorescence microscope (Leica Camera, Wetzlar, Germany) and a Zeiss Axiovert LSM 710 laser scanning confocal microscope (Carl Zeiss, Oberkochen, Germany).

*Western Blot:* Rabbit anti-FAAH (generous gift from Ken Mackie, 1:500), mouse anti-HA (Covance, Dedham, MA, USA, 1:500), and rabbit anti-actin (Chemicon, Temecula, CA, USA, 1:3000) were used as primary antibodies, and anti-rabbit and anti-mouse horseradish peroxidase (Dianova, Hamburg, Germany, 1:5000) as secondary antibodies. Signal was detected by Amersham ECL Prime Western Blotting Detection Reagent (GE Healthcare Life Sciences). Quantification was performed with Bio 1D.CAPT Software (Vilber Lourmat, Eberhardzell, Germany). Densitometric data were normalized to actin.

*Endocannabinoid Extraction and Liquid Chromatography–Mass Spectrometry:* Extraction of eCBs from cells: To prevent alterations of lipid levels, all extraction steps were performed at 4°C. Transfected cells were harvested and centrifuged for 10 min (2000g, 4°C). Supernatant was spiked with 500 μl ethylacetate/hexane (9:1, v/v) containing the internal standards to a target concentration (50 μl final volume) of 0.5 ng/ml AEA-d4, 50 ng/ml 2-AG-d5, 100 ng/ml AA-d8, 2 ng/ml OEA-d2 and 2 ng/ml PEA-d4 (BIOMOL Research Laboratories Inc.). Samples were homogenized with the Disruptor Genie (Scientific Industries, Inc., Bohemia, NY, USA) for 30 sec, centrifuged for 15 min. (16000g, 4°C) and frozen for 10 min. Cell pellets were resuspended in 50 μl Spike Solution and 500 μl ethylacetate/n-hexan (9:1 v/v). Pre-cooled 1.4mm Zirconium oxide beads (Precellys 24, Bertin Technologies, Montigny-le Bretonneux, France) were added to each sample and homogenized for 15 sec. with 5000 cycles with the Disruptor Genie (Scientific Industries, Inc., Bohemia, NY, USA). 200 μl ice-cold 0.1 M formic acid was added to each sample and centrifuged at 8000 g for 10 min at 4°C. The upper organic phase was recovered and evaporated under a gentle stream of nitrogen at 37°C and reconstituted in 50 μl acetonitrile/water (1:1; v/v). Samples were stored at -20°C till use. Extraction of eCBs from brain tissue: Hippocampi were isolated and transferred into extraction tubes containing 7 steel balls (ø2.5 mm) and 300 μl ethylacetate/hexane (9:1, v/v) containing the internal standards with 300μl of 50 mM Tris buffer, 150 mM NaCl, and Halt Protease/Phosphatase inhibitor (Thermo Scientific). Tissue was homogenized with a TissueLyser II (Qiagen, Venlo, Netherlands) at 30 Hz for 1 min, centrifuged at 16000 g at 4°C for 15 min and kept on dry ice (10 min) to freeze the aqueous phase. The upper organic phase was evaporated under a gentle stream of nitrogen at 37°C using a vapotherm (Barkey, Leopoldshöhe, Germany). The dry lipid extracts were dissolved in 100 μl acetonitrile/water (1:1), vortexed for 30 s and centrifuged at 10000 g, 3 min at 4°C. 50 μl of samples were pipetted into a glass insert placed in a glass vial and closed with a slitted lid cap. The precooled (4°C) CTC HTC PAL autosampler (CTC Analytics AG, Zwingen, Switzerland) was loaded with vials containing samples or calibration standards. AEA, PEA, OEA, 2-AG, and AA lipid concentrations were measured and quantified as previously described (Bindila and Lutz, 2016).

*FAAH activity assay:* FAAH activity assay was based on the hydrolysis of [^3^H]-AEA by FAAH, resulting in [3H-ethanolamine] and arachidonic acid. Hippocampi were dissected and frozen at -80°C until use. Tissue was homogenized by mechanical disruption with a glass homogenizer in buffer containing 50 mM Tris-HCl, pH 7.4, 1 mM EDTA and protease inhibitors (complete cocktail tablets, Roche) and spun at 1000 g for 5 min at 4°C to remove debris. Protein concentrations were measured via BCA Protein Assay Kit (Thermo Fisher Scientific, MA, USA). Hippocampal homogenates (38 μg protein per reaction) were diluted in homogenization buffer containing 0.2% bovine serum albumin (BSA) and FAAH substrate [^3^H]-AEA (American Radiolabeled Chemicals, specific activity: 60 Ci/mmol; concentration: 1 mCi/ml) in a final volume of 500 μl. Due to the low concentration of the radiolabeled substrate, [^3^H]-AEA was diluted by adding unlabeled AEA (5 mg/100 μl, Cayman Chemicals) in order to generate six concentrations (0-50 μM range) with the highest concentration containing 50 μM of unlabeled AEA and 0.5 nM of [^3^H]-AEA. Background was measured in presence of the FAAH inhibitor arachidonoyl serotonin (AA-5HT, 25 μM; Cayman Chemicals). Blank samples containing [^3^H]-AEA without proteins were used as additional background measurement. After an incubation of 30 min at 37°C, the reaction was stopped by adding 800 μl of chloroform/methanol (1:2) followed by centrifugation at 3000 g for 5 min at room temperature. 800 μl of the upper aqueous phase containing the product [^3^H]-ethanolamine was counted in a liquid scintillation β-counter for radioactivity quantification. Specific values were determined by subtracting blank (absence of proteins) and background values (presence of AA-5HT). Three independent experiments (n=3 in duplicate for each AEA concentration) for each experimental group (AAV-WT and AAV-Glu-FAAH) were carried out. Data are expressed in pmol/(min*mg), referring to the amount of product [3H-ethanolamine]; min refers to the reaction time (30 min); mg refers to the amount of protein used (0.038 mg).

*Electrophysiology:* Mice were decapitated under deep isoflurane anesthesia (1-chloro-2,2,2-trifluoroethyl difluoromethyl ether; 2.5% in O_2_; Abbot GmbH & Co. KG, Germany), and brains were quickly removed. Horizontal slices (300 μm) containing the hippocampus and the entorhinal cortex were obtained with a Leica VT1200S vibratome (Leica Microsystems CMS, Mannheim, Germany). The preparation was performed in ice-cold oxygenated (95% O_2_, 5% CO_2_), saccharose-based artificial cerebrospinal fluid (ACSF) containing (in mM): KCl 2.5, Na_2_PO_4_ 1.25, PIPES 20, CaCl_2_ 0.5, MgSO_4_ 10, saccharose 200, glucose 10 at a pH of 7.35. After preparation, slices were kept under submerged conditions at 30°C for approximately 30 min in preincubation ACSF (in mM: NaCl 125, Na_2_PO_4_ 1.25, NaHCO_3_ 24, KCl 2.5, CaCl_2_ 2, MgSO_4_ 2, glucose 10 at a pH of 7.35) and were then transferred to physiological ACSF solution (in mM: NaCl 120, Na_2_PO_4_ 1.25, NaHCO_3_ 22, KCl 2.5, CaCl_2_ 2, MgSO_4_ 2, glucose 25 at a pH of 7.35) at room temperature for further storage.

Whole-cell patch-clamp recordings in voltage-clamp mode were obtained from CA1 pyramidal cells under submerged conditions at near physiological temperatures (32-34°C). For characterization of intrinsic discharge and membrane properties, hyper- and depolarizing current steps (50 pA, 500 ms) were used. Input resistance (R_in_) and membrane time constant (τ) were calculated at a 100 pA hyperpolarizing current step. The input resistance was calculated by: R_in_=ΔV/I. ΔV was measured under steady-state conditions at the end of the injected hyperpolarizing current pulse. τ was obtained by a monoexponential fit of the membrane potential shift induced by the hyperpolarizing current step. Sag ratio was calculated by dividing the steady-state voltage during a hyperpolarizing current pulse of 200 pA by the peak voltage during hyperpolarization. Action potential (AP) properties were assessed from the first AP elicited by a 500 ms lasting depolarizing current injection step at -70 mV. The membrane potential at which dV/dt reached a criterion of 10 mV/ms was used to estimate AP threshold. AP height was defined as the amplitude between AP threshold and peak of the AP. AP half-width was measured at 50 % AP height. Postsynaptic currents (PSCs) were evoked by a bipolar tungsten stimulation electrode placed locally in stratum radiatum. 2-(3-Carboxypropyl)-3-amino-6-(4 methoxyphenyl) pyridazinium bromide (Gabazine, 10 μM), CGP55845 hydrochloride (2.5 μM), DL-2-amino-5-phosphonopentanoic acid sodium salt (AP5, 25 μM), or 6,7-dinitroquinoxaline-2,3-dione disodium salt (DNQX, 10 μM) were added to the extracellular solution as required to block GABAergic or glutamatergic postsynaptic currents, and to isolate postsynaptic excitatory (EPSCs) or inhibitory (IPSCs) currents. Patch-clamp electrodes (2-4 MΩ) were made of borosilicate glass (GC150T-10, Harvard Apparatus, UK). Access resistance was monitored throughout the experiments and did not exceed 25 MΩ. Recordings with higher or fluctuating access resistance were discarded. No series resistance compensation was used. Electrophysiological data were sampled at 5-10 kHz with an EPC10-double amplifier (HEKA, Germany) and analyzed offline with Clampfit software (Molecular Devices Corporation, Sunnyvale, CA, USA). For analysis, evoked PSCs were normalized to baseline values.

In short-term plasticity experiments, cells were clamped at a holding potential of -70 mV and the intracellular solution contained (in mM): K-gluconate 100, KCl 50, CsCl 10, HEPES 10, EGTA 0.2, MgCl_2_ 1, Mg-ATP 1, and Na-GTP 0.3, adjusted with KOH to a pH of 7.30. After recording of stable baseline responses (stimulation frequency 0.2 Hz), depolarization-induced suppression of excitation (DSE) or inhibition (DSI) was tested by application of a depolarization step (−70 to 0 mV, 10 s). The mean of 5 electrically evoked responses immediately before the depolarization (pre) was compared with 5 evoked responses recorded immediately after depolarization (post I), 5 responses after 2 min (post II) and 5 responses 4 min after depolarization (post III). Statistical significance between groups was tested using two-way repeated measures analysis of variance (RM-ANOVA) with time and group (AAV-WT or AAV-Glu-FAAH) as factors followed by post hoc Sidak’s multiple comparison test.

In long-term plasticity experiments, PSCs were evoked at a frequency of 0.033 Hz. Stable baseline responses were recorded for 10 min. In case of long-term potentiation (LTP) experiments and for recording of spontaneous (s) EPSCs, cells were held at -70 mV and the intracellular solution contained (in mM): K-gluconate 135, KCl 20, HEPES 10, phosphocreatine 7, Mg-ATP 2, Na-GTP 0.3, EGTA 0.2, adjusted with KOH to a pH of 7.2. The stimulus intensity was set to evoke EPSC amplitudes of 30–50% of the maximum response during baseline recordings. LTP was induced by application of 4 trains of 100 pulses at 100 Hz with 30 s inter-train intervals. In excitatory LTD (eLTD) experiments, ACSF with 1 mM MgSO_4_ was used. Cells were held at -70 mV and the stimulus intensity was set to evoke EPSC amplitudes of 50% of the maximum response during baseline recordings. eLTD was induced by application of 900 paired pulses (50 ms inter-pulse interval) at 1 Hz (Kemp and Bashir, 1997; Kemp *et al*, 2000). In inhibitory long-term depression (iLTD) experiments and for recording of spontaneous (s) IPSCs, neurons were held at +10 mV and the recording pipette was filled with (in mM): Cs-methanesulfonate 133, NaCl 20, MgCl_2_ 1, CaCl_2_ 0.3, EGTA 1, HEPES 10, Mg-ATP 2, Na-GTP 0.3, glucose 10, adjusted with CsOH to a pH of 7.3. The stimulus intensity was set to evoke IPSC amplitudes of 50–70% of the maximum response during baseline recordings. iLTD was induced by application of 2 trains of 100 pulses at 100 Hz with 20 s inter-train intervals (Chevaleyre and Castillo, 2003). Changes in synaptic strength were measured for 30 min after induction. Long-term plasticity was calculated by averaging the responses collected during min 36-40 (LTP or iLTD) or min 46-50 (eLTD) of each experiment. All individual cells recorded were included into analysis and no arbitrary threshold was set to define LTP, eLTD or iLTD. Statistical significance between groups (AAV-WT and AAV-Glu-FAAH) was tested using Student’s test. Sample traces are averages of 5 to 10 responses, stimulus artefacts are clipped.

Paired-pulse index (PPI) was investigated by analyzing the ratio of the second to the first synaptic response (EPSC2/EPSC1 or IPSC2/IPSC1) at an inter-stimulus interval of 50 ms (EPSCs) or 100 ms (IPSCs). Statistical significance between groups was tested using two-way RM-ANOVA with time (pre vs post) and group (AAV-WT or AAV-Glu-FAAH) as factors followed by post hoc Sidak’s multiple comparison test. The coefficient of variation (CV) was calculated as CV-index: CV^-2^ = (mean of EPSC/SD of EPSC)^2^ or CV^-2^ = (mean of IPSC/SD of IPSC)^2^ from a stable recording period within the last 10 minutes of baseline and LTP or iLTD, respectively. Changes in CV^-2^ were analyzed using paired Student’s test.

Detection and analysis of sEPSCs and sIPSCs was done offline using Mini Analysis program (Version 6.0.7, Synaptosoft Inc., Fort Lee, NJ, USA). Detection threshold for amplitude of spontaneous events was set 3 times above root mean square (RMS)-baseline noise level. The baseline noise level was quantified from a recording episode without phasic synaptic currents using the RMS analysis routines packaged with the Mini Analysis program. All events were visually controlled to exclude false-positive events. 1 to 5 min lasting recording episodes were analyzed. 50 consecutive events of each cell were randomly chosen for generation of cumulative histograms and differences in distributions were statistically tested using Kolmogorov-Smirnov test in GraphPad Prism (Version 6.01, GraphPad Software, La Jolla, CA, USA). To analyze rise and decay times (T_rise_ and T_decay_), 50 randomly chosen events of each cell that lacked large-amplitude spontaneous currents during the decay phase were averaged for each cell. T_rise_ and T_decay_ were defined as the time between 10 and 90% of the rising phase and the time between 90 and 37% of the decaying phase of the average sEPSC or sIPSC peak amplitude, respectively, and obtained by fitting the decay phase to a biexponential function using the curve fitting tool packaged with the Mini Analysis program.

**Supplementary References**

Bindila L, Lutz B (2016). Extraction and Simultaneous Quantification of Endocannabinoids and Endocannabinoid-Like Lipids in Biological Tissues. Methods Mol Biol 1412, 9-18.

Chevaleyre V, Castillo PE (2003). Heterosynaptic LTD of hippocampal GABAergic synapses: A novel role of endocannabinoids in regulating excitability. *Neuron* **38**: 461–472.

Kemp N, Bashir ZI (1997). NMDA receptor-dependent and -independent long-term depression in the CA1 region of the adult rat hippocampus in vitro. Neuropharmacology 36: 397–9.

Kemp N, McQueen J, Faulkes S, Bashir ZI (2000). Different forms of LTD in the CA1 region of the hippocampus: role of age and stimulus protocol. Eur J Neurosci 12: 360–6.

**Supplementary Figure 1**


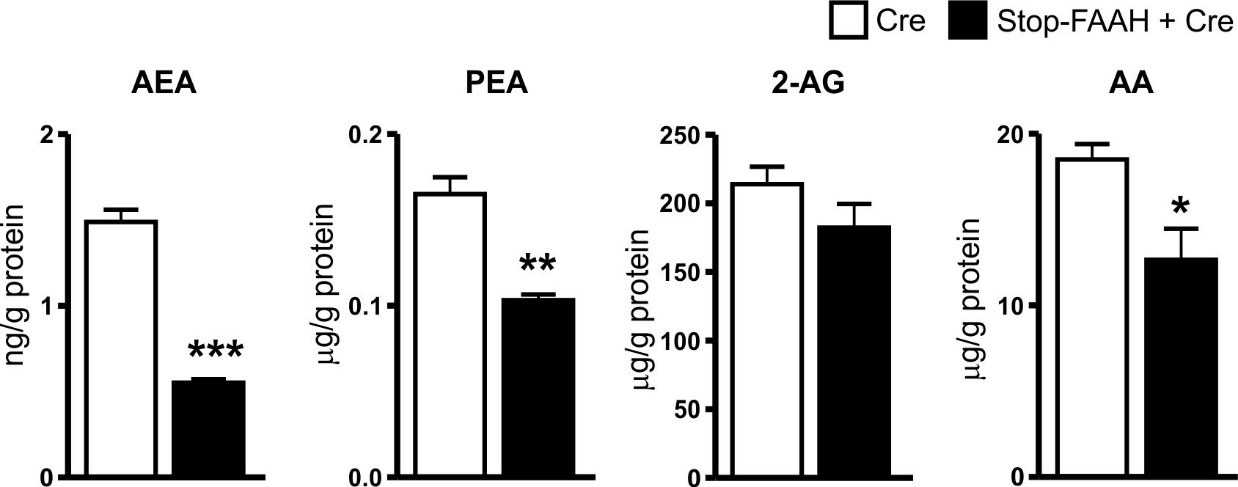


**Supplementary Figure S1:** Overexpression of fatty acid amide hydrolase (FAAH) in HEK293 cells leads to increased degradation of anandamide (AEA) and palmitoylethanolamide (PEA). LC-MS/MS analysis of FAAH-overexpressing HEK293 cells revealed decreased levels of AEA, PEA, and AA levels 60 hours after co-transfection of Stop-FAAH and Cre recombinase expression plasmids as compared to control cells, containing only Cre recombinase expression plasmid. 2-AG levels were not significantly altered. *p<0.05, **p<0.01, ***p<0.001, Student´s t-test. Data are represented as mean (n=6) ± SEM.

**Supplementary Figure 2**


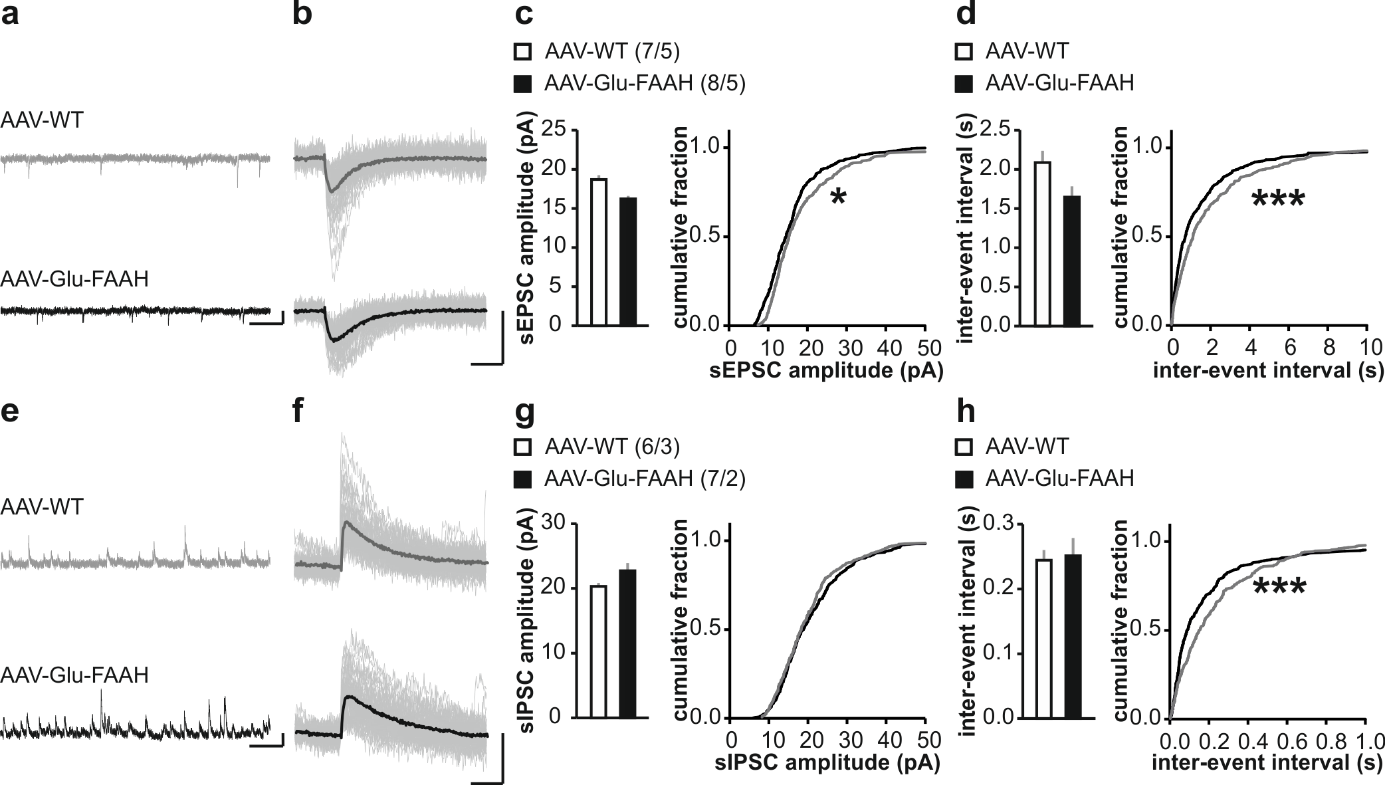


**Supplementary Figure S2:** FAAH overexpression in hippocampal CA1-CA3 pyramidal neurons increases spontaneous glutamatergic and GABAergic transmission at CA3-CA1 synapses. **(a-d)** Spontaneous glutamatergic synaptic transmission at hippocampal CA3-CA1 synapses. **(a)** Example traces of sEPSCs recorded in CA1 pyramidal cells from AAV-WT (top, grey) and AAV-Glu-FAAH (bottom, black) mice. Scale bars, 20 pA, 1 s. **(b)** Exemplary averaged time courses of sEPSCs (black lines) derived from 50 events (grey lines) from the recordings of cells shown in (a). Scale bars, 20 pA, 10 ms. **(c)** Cumulative histogram of sEPSC amplitudes reveals slightly smaller amplitudes in AAV-Glu-FAAH mice (Kolmogorov–Smirnov test, *p<0.05). **(d)** Cumulative histogram of sEPSC inter-event intervals reveals decreased inter-event intervals in AAV-Glu-FAAH mice (Kolmogorov–Smirnov test, ***p<0.001). **(e-h)** Spontaneous GABAergic synaptic transmission at hippocampal CA3-CA1 synapses. **(e)** Example traces of sIPSCs recorded in CA1 pyramidal cells from AAV-WT (top, grey) and AAV-Glu-FAAH (bottom, black) mice. Scale bars, 20 pA, 500 ms. **(f)** Exemplary averaged time courses of sIPSCs (black lines) derived from 50 events (grey lines) from the recordings of cells shown in (e). Scale bars, 20 pA, 10 ms. **(g)** Cumulative histogram of sIPSC amplitudes in AAV-WT and AAV-Glu-FAAH mice (Kolmogorov–Smirnov test, p=0.19). **(h)** Cumulative histogram of sIPSC inter-event intervals reveals decreased inter-event intervals in AAV-Glu-FAAH mice (Kolmogorov–Smirnov test, ***p<0.001). In (c, d, g, h), columns represent mean ± SEM of inter-event intervals or amplitudes plotted in cumulative histograms. Numbers indicate the number of recorded cells/animals. 50 events per cell were analyzed.

**Supplementary Table S1.** Passive and active membrane properties of CA1 pyramidal neurons.

|  | AAV-WT | AAV-Glu-FAAH | unpaired Student’s t test |
| --- | --- | --- | --- |
| resting membrane potential (mV) | -67 ± 2 | -68 ± 1 | p=0.76 |
| input resistance (MΩ) | 53 ± 6 | 52 ± 6 | p=0.87 |
| membrane time constant (τ in ms) | 14.8 ± 1.2 | 16.8 ± 1.7 | p=0.38 |
| AP threshold (mV) | -41 ± 2 | -41 ± 2 | p=0.92 |
| AP height (mV) | 96 ± 4 | 96 ± 3 | p=0.99 |
| AP half-width (ms) | 1.02 ± 0.04 | 1.04 ± 0.03 | p=0.83 |
| sag ratio | 0.80 ± 0.02 | 0.82 ± 0.01 | p=0.48 |
| cells/animals | 7/5 | 8/5 |  |

**Supplementary Table S2.** Statistical Data Analyses (referred to Figure 1f and Figure 5a-k)





Unpaired Student's *t*-test of results shown in Figure 1f (western blot) and Figure 5 (behavior). Statistical analysis results (df, degree of freedom; t; R2, R-squared; *p* values) and N number are shown and statistical significant values are reported in bold. Statistical results are shown as asterisks in the corresponding figures.

**Supplementary Table S3.** Statistical Data Analyses (referred to Figure 2)

**
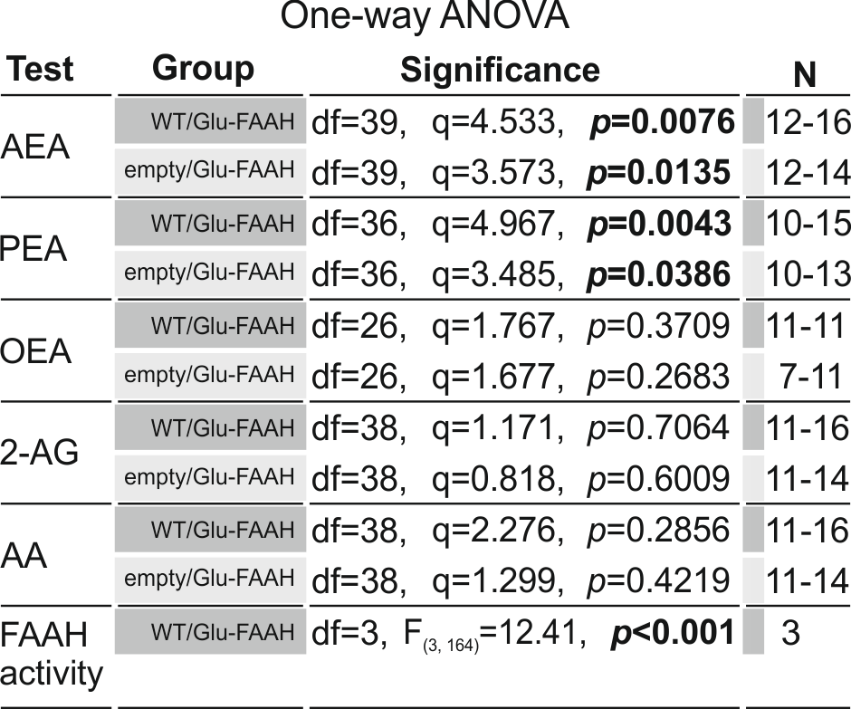
**

One-way ANOVA of results (lipid measurements, FAAH activity) shown in Figure 2. Statistical analysis results (F, df, degree of freedom; q and *p* values) and N number are shown and statistical significant values are reported in bold. Results were further analyzed by Tukey's post-hoc test to identify differences between group means. Statistical results from post-hoc analysis are shown as asterisks in Figure 2. Abbreviations: AEA, anandamide; PEA, palmitoylethanolamide; OEA, oleoylethanolamide; 2-AG, 2-arachidonoylglycerol; AA, arachidonic acid.

**Supplementary Table S4.** Statistical Data Analyses (referred to Figure 5l)

**

**

Two-way ANOVA repeated measures of results shown in Figures 5l (passive avoidance). The effects of ‘time’ and ‘genotype’ (F; df, degree of freedom; t and *p* values) and N number are shown and statistical significant values are reported in bold. ‘Time’ refers to the time points when the passive avoidance test was carried out and is the repeated factor. Results were further analyzed by Sidak’s post-hoc test to identify differences between group means. Statistical results from post-hoc analysis are shown as asterisks in Figure 5l. Abbreviations: Acq, acquisition; h, hour; w, week.
